# Typhoid and Tuberculosis

**Published:** 1914-06

**Authors:** 


### Typhoid and Tuberculosis.

The predisposition to tuberculosis due to typhoid, independently suggested by Reincke of Hamburg and Mills of this country in 1907, has received considerable attention recently. The **Boston M. and S. Jour.**, Feb. 12, 1914, page 259, calls attention to the parallelism of the mortality curves of these two diseases. It occurs to us that there are reasons why testimony, even statistic, on this relation of two infections, should be regarded with scepticism. Tuberculosis has, for many years accounted for just about 10% of the total mortality, the curve declining for each so as to maintain this ratio. Excluding deaths in infancy and traumatic deaths, nearly a fifth of the total human material liable to death from various diseases, actually die of tuberculosis. When so large a proportion of the population is involved, only a marked difference from average incidence and mortality can be considered significant.

The parallelism between the curves of the two diseases can be well explained by the fact that both depend on crowding and by hygiene and sanitation, due to the development of civilization, and both are limited by a higher civilization which meets the problems presented by itself..

Typhoid is one of the diseases in which general health and resistance seem to have little bearing on susceptibility. Tuberculosis, on the other hand is almost the only infectious process in which susceptibility plainly does depend on these factors. (Before denying this statement, check off the list of infections.) It must be admitted that, unless there is a specific opposition of bacteria, any such sickness as typhoid predisposes in a general way to tuberculosis, not to mention the fact that it is only recently that the typhoid patient, seeking aid in a hospital, even in a private room, has undergone an abnormally high degree of exposure to tubercular fomites. Whether, with these qualifications, a genuine predisposition to tuberculosis is conferred by typhoid, is a question to be decided by a sceptic study of the evidence.

